# A Co-culture Model of PBMC and Stem Cell Derived Human Nasal Epithelium Reveals Rapid Activation of NK and Innate T Cells Upon Influenza A Virus Infection of the Nasal Epithelium

**DOI:** 10.3389/fimmu.2018.02514

**Published:** 2018-11-08

**Authors:** Annika Luukkainen, Kia Joo Puan, Nurhashikin Yusof, Bernett Lee, Kai Sen Tan, Jing Liu, Yan Yan, Sanna Toppila-Salmi, Risto Renkonen, Vincent T. Chow, Olaf Rotzschke, De Yun Wang

**Affiliations:** ^1^Department of Otolaryngology, Yong Loo Lin School of Medicine, National University of Singapore, Singapore, Singapore; ^2^Haartman Institute, University of Helsinki, Helsinki, Finland; ^3^Singapore Immunology Network (SIgN), A^*^STAR (Agency for Science, Technology and Research), Singapore, Singapore; ^4^Skin and Allergy Hospital, University of Helsinki and Helsinki University Hospital, Helsinki, Finland; ^5^HUSLAB, Helsinki University Hospital, Helsinki, Finland; ^6^Department of Microbiology and Immunology, Yong Loo Lin School of Medicine, National University of Singapore, Singapore, Singapore

**Keywords:** influenza A virus, peripheral blood mononuclear cells, nasal epithelium, co-culture, innate T cells

## Abstract

**Background:** We established an *in vitro* co-culture model involving H3N2-infection of human nasal epithelium with peripheral blood mononuclear cells (PBMC) to investigate their cross-talk during early H3N2 infection.

**Methods:** Nasal epithelium was differentiated from human nasal epithelial stem/progenitor cells and cultured wtih fresh human PBMC. PBMC and supernatants were harvested after 24 and 48 h of co-culture with H3N2-infected nasal epithelium. We used flow cytometry and Luminex to characterize PBMC subpopulations, their activation and secretion of cytokine and chemokines.

**Results:** H3N2 infection of the nasal epithelium associated with significant increase in interferons (IFN-α, IFN-γ, IL-29), pro-inflammatory cytokines (TNF-α, BDNF, IL-3) and viral-associated chemokines (IP-10, MCP-3, I-TAC, MIG), detectable already after 24 h. This translates into rapid activation of monocytes, NK-cells and innate T-cells (MAIT and γδ T cells), evident with CD38+ and/or CD69+ upregulation.

**Conclusions:** This system may contribute to *in vitro* mechanistic immunological studies bridging systemic models and possibly enable the development of targeted immunomodulatory therapies.

## Introduction

Seasonal influenza A viruses (IAV) account for 3 to 5 million cases of severe illness and an estimated 250,000 to 500,000 deaths annually worldwide (1). Seasonal IAV infections range from mild to severe and life-threatening forms, including acute respiratory distress syndrome. Children under 2 years of age, patients with chronic conditions and adults older than 65 are risk groups for increased morbidity and mortality due to influenza infection (1). IAV first infects the upper airways, nose and pharynx, from where it can spread to the lower airways in severe cases (1). Seasonal IAV infects epithelial cells by binding to alpha 2–6 sialylated glycans on the surface of upper airway epithelial cells with its surface protein hemagglutinin whilst progeny virions are released from infected host cells by neuraminidase activity (2).

Both innate and adaptive immunity play critical roles in the host defense against influenza viruses. Whilst the adaptive immune response is important in viral clearance and mediating protective immunity against IAV, the innate immune response can influence the outcome of the adaptive immune response (3). The innate immune response mediated through innate sensors is required to block viral replication and promote viral clearance. In order to prevent re-infection, a strain-specific humoral response to influenza is necessary, whereas viral clearance is mediated by CD8+ T cell responses (4, 5).

The first days of seasonal IAV infection have yet to be documented in as much detail as invasive influenza or resolution of IAV infection. *In vitro* co-culture models involving airway epithelial cell lines or bronchial epithelium with immune cells have already been investigated. However, most co-culture studies have been conducted on cell lines whilst upper airway epithelium and co-cultures with upper airway epithelium have yet to be studied. Therefore, to address this gap in knowledge, we have previously established a human nasal epithelium derived from human nasal epithelial stem/progenitor cells (hNESPC) model and H3N2-infection of the nasal epithelium (6, 7).

The aim of this study was to extend this approach in order to establish a human model of the nasal mucosa which allowed the investigation of epithelium-leukocyte cross-talk during early H3N2 infection. For this we established a contact free co-culture model in which hNESC-derived nasal epithelium were cultured on Transwell inserts while freshly isolated peripheral blood mononuclear cells (PBMC) were cultured in the chamber underneath. The physical separation prevented the direct infection of the PBMC and thus allowed us to study the response of the immune cells triggered by soluble factors released by the infected epithelial layer by flow cytometry and Luminex.

## Methods

### Ethical aspects

Ethical approval to conduct this study was obtained from the National Healthcare Group Domain-Specific Board of Singapore (DSRB Ref: D/11/228) and Institutional Review Board of the National University of Singapore (IRB code 13-509) in accordance with the Helsinki declaration. Written informed consent was obtained from all study subjects prior to sample collection. The demographics of the donors' PBMC are summarized in Table 1A.

**Table 1A:** Nasal epithelium donor characteristics.

**Sex**	**Age**	**Atopy**	**Other conditions**	**Medication**	**Ethnicity**
Male	60	Yes	Non-smoker, asthma	inGC treatment	Chinese

*inGC, intranasal glucocorticoid*.

### Study participants

#### Human nasal epithelium

Human nasal epithelial cells (hNEC) were derived from hNESPCs isolated from biopsies from a patient with chronic rhinosinusitis with nasal polyps undergoing surgery at the National University Hospital, Singapore. The donor characteristics are presented in Table 1A. The subject was free of symptoms of upper respiratory tract infection before surgery. hNESPC cell culture methods to normalize sample to its native state through stem cell enrichment and passaging have been described (6, 8, 9). Briefly, the hNESPCs were transferred to an air-liquid interface system to become fully differentiated hNECs cells within 4 weeks. The hNECs were characterized by means of immunofluorescence staining and 3-D confocal imaging of ciliated and goblet cell markers (i.e., mouse anti-human βIV-tubulin, clone ONS.1A6, ab11315; Abcam, Cambridge, Mass; and rabbit anti-human mucin5AC, sc-20118; Santa Cruz Biotechnology, Dallas, Tex). In our co-culture assays, we either seeded hNECs at a density of 1.5 × 10^5^ cells per well in 24 well plate or 3 × 10^5^ cells per well in 12 well plate transwells. Transwells have a pore size of 0.4 μm.

Epithelial integrity was measured by transepithelial electrical resistance (TEER) in four randomly chosen Transwells. TEER measurements were performed using an EVOM voltohmmeter device (WPI, Sarasota, FL, USA). Briefly, 0.5 and 1.0 mL of pre-equilibrated medium were added to the apical and basal chambers. Measurements were performed after obtaining a steady signal for 5 min in the blank insert, and corrected by subtracting the background of the blank transwell inserts and medium-only inserts. The final TEER reading is presented in Ω.cm2 (TEER measurment × area of membrane).

### Peripheral blood mononuclear cells and isolated NK cells

Whole blood was collected in 9 ml K_3_EDTA Vacutainer Tubes (Greiner Bio-One, Kremsmünster, Austria). Peripheral blood mononuclear cells (PBMC) were isolated from fresh venous blood taken from 5 healthy adult volunteers at the National University of Singapore and at the Singapore Immunology Network, Agency for Science Technology and Research (Table 1B). PBMC were isolated from the buffy coat by density gradient centrifugation with Lymphoprep (Stemcell, Axis-Shield PoC AS, Oslo, Norway), followed by PBS washes until the supernatant was clear. We obtained a 98% viability of PBMC, as assessed by Trypan blue staining. PBMC were suspended at a density of 4 × 10^6^/ml in complete media (RPMI supplemented with 10% heat-inactivated FBS, 1% L-glutamine, 1% penicillin-streptomycin). One of the PBMC donors had to be excluded after quality control *post-hoc* analyses. NK cells were isolated by negative selection from PBMC using EasySEp Human NK Cell Enrichment Kit (Stemcell Technologies). The purity of the isolated NK cells was 84.4% of CD45+ cells.

**Table 1B:** PBMC donor characteristics.

**Donor**	**Sex**	**Age**	**Atopy**	**Other conditions**	**Medication**	**Ethnicity**
Donor 1	M	46	+	–	–	Chinese
Donor 2	F	45	+	–	–	Chinese
Donor 3	F	30	+	–	–	Chinese
Donor 4	F	26	+	–	–	Chinese

*M, male; F, female; atopy status: atopic, +; non-atopic, −*.

### IAV and co-culture

**Figure 1:**
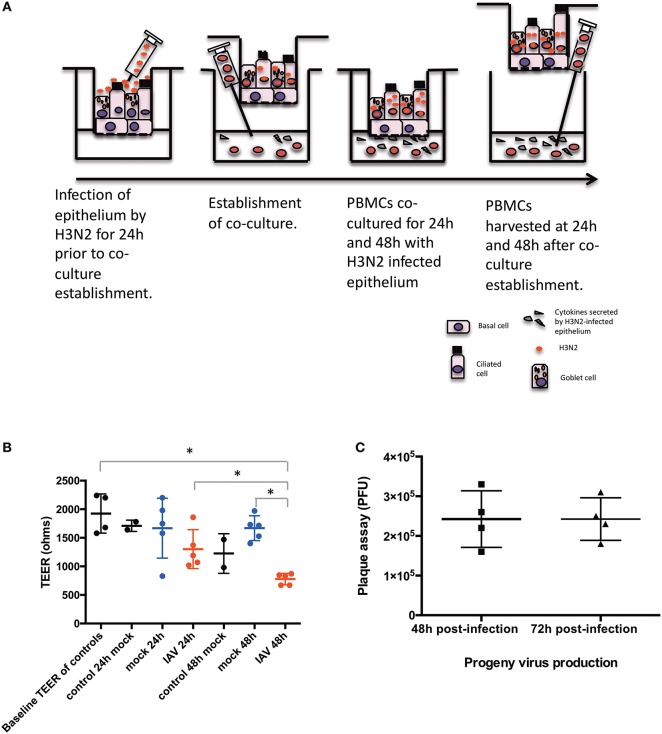
Diagram of the conditions of the study. **(A)** Time-points and settings of the co-culture. **(B)** Transepithelial electrical resistance (TEER) measurements post co-culture establishment. Control, hNEC only; mock, hNEC with PBMC in RPMI; IAV, IAV-infected hNEC with PBMC in RPMI. **(C)** Progeny viruses detected by viral plaque assay from apically applied H3N2 inoculum. ^***^P < 0.001; ^**^P < 0.01; ^*^P < 0.05; NS, not significant.

hNECs were grown on Transwell inserts for an average of 26 days. hNECs were infected by Influenza A virus (IAV) Aichi/2/1968 (H3N2) (ATCC) for 24 h prior to formation of the co-culture with PBMC. H3N2 was diluted from a stock concentration of 9.0 × 10^5^ PFU/100 μL to an MOI of 0.1 (10^4^ PFU/insert) in fully complemented RPMI. An MOI of 0.1 of H3N2 was applied apically to the fully differentiated nasal epithelium. After 24 h of H3N2 infection of the hNECs, PBMC in complete media were seeded immediately following density gradient isolation at the bottom of the Transwells at a concentration of 1.5 x 10^6^ cells in each well for the 24 well plates or 3 × 10^6^ cells for the 12 well plates. Cells were kept in co-culture for 24 and 48 h (48 and 72 h post infection). The diagram of the co-culture setup is presented in Figure 1A.

We used the following terms to define treatment of the epithelium. Direct infection of the epithelium was defined as IAV infection. Mock infection was defined as apical application of the same amount of complete media without IAV to nasal epithelium (mock). PBMC-only refers to only PBMC cultured without nasal epithelium.

### Antibodies and flow cytometry

All antibodies used in this study were purchased from BD Biosciences unless stated otherwise. The clone numbers are indicated in parentheses. PBMC were harvested for flow cytometry after 24 and 48 h post-establishment of the co-culture. Cells were first washed with PBS and incubated at room temperature for 15 min with Fixable Live/Dead Blue Viable Dye (Thermo Scientific). After incubation, the cells were washed in PBS buffer containing 0.5% BSA and 2 mM EDTA. Subsequently, the cells were incubated with the following antibodies: Vδ1 TCR (TS-1, Thermo Scientific) FITC, Vδ2 TCR (B6, Biolegend) PerCP, CD3 (UCHT1) V500, CD8 (SK1) APC-Cy7, CD14 (MϕP9) PE-CF594, CD56 (B159) PE-Cy7, CD69 (FN50, Biolegend) BV421, CD83 (HB15e) APC, CD161 (HP-3g10, Biolegend) BV605, Vα7.2 (3C10, Biolegend) PE, and CD38 (HB7) BUV395 for 15 min at 4°C. Assays on NK cells were performed additionally with the following antibodies: CD3 (UCHT1) V500, CD16 (3G8, Biolegend) FITC, CD56 (B159) PE-Cy7, CD38 (HB7) BUV395, CD69 (FN50 Biolegend) PE. Flow cytometry analyses were performed using a BD LSRII flow cytometer (BD). Subsequent gating analyses were performed using FlowJo software version 9.9.6 or 10.5.2 (Tree Star Inc.).

### Cytokines and chemokines

Basal media supernatant of the co-culture after PBMC harvesting was also collected at 24 and 48 h post co-culture establishment and immediately stored at −80°C. 25 μL of culture supernatant were used in the measurement of Milliplex MAP Kits (23-Plex Human Cytokine/Chemokine Panel). The cytokines and chemokines studied included IFN-α, IFN-γ, IL-29, GM-CSF, G-CSF, TSLP, MCP-1 MCP-3, BDNF, MIG, IP-10, I-TAC, SDF-1α, TNF-α, IL-1α, IL-1β, IL-2, IL-3, IL-4, IL-5, IL-6, IL-8 and IL-12p40. The concentration of each analyte was calculated using the Bio-Plex manager 6.0 software with a 5-parameter curve fitting algorithm applied for standard curve.

### Viral RNA

Viral RNA was extracted from the stock H3N2 solution, the MOI 0.1 H3N2 aliquot used in the assays, 10-times serial dilutions of the MOI 0.1 H3N2 aliquot and basal medium from all samples with IAV infection at both 24 and 48 h after co-culture establishment. RNA was extracted with the viral RNA extraction kit (Qiagen). Following RNA extraction, RNA was immediately converted to cDNA (Thermo Fisher Scientific, Waltham, MA, USA). Non-structural gene (NS1) and matrix (M1) were selected as targets for PCR. We used primers with the following sequences: NS1 Forward 5′-AGCAAAAGCAGGGTG; Reverse 5′-TATTAGTAGAAACAAGGGTGTTTT, M1 Forward 5′-GGAGCAAAAGCAGGTAG; Reverse 5′-ATTAGTAGAAACAAGGTAGTTTTT (Sigma). We used real-time quantitative PCR to measure NS1 and M1 mRNA levels, and correlated them with the standard curve from the MOI 0.1 virus stock serial dilution. Real-time quantitative PCR was performed in technical triplicates for all samples.

### Plaque assay

The apical RPMI medium was collected upon 24 and 48 h after co-culture establishment for viral quantification. MDCK cells at 85–95% confluence in 24-well plates were incubated with 100 μL of serial dilutions from 10^−1^-10^−4^ of virus from infected hNECs at 35°C for 1 h. The plates were rocked every 15 min to ensure equal distribution of virus. The inoculums were removed and replaced with 1 mL of Avicel (FMC Biopolymer) overlay on each well and incubated at 35°C with 5% CO_2_ for 65–72 h. Avicel overlay was removed after the incubation period and cells were fixed with 4% formaldehyde solution in 1 × PBS for 1 h. Formaldehyde solution was removed and cells were washed with 1 × PBS. The fixed cells were stained with 1% crystal violet for 15 min and washed with running water. The plaque-forming units (PFU) were calculated as follows: Number of plaques × dilution factor = number of PFU per 100 μL.

### Statistics

Statistics were performed with Spotfire (version 6.5.2) and SPSS 22.0 statistical software package (IBM SPSS Statistics for Windows, Armonk, NY: IBM Corp. Released 2013). Comparisons were performed by a 2-way ANOVA between groups (IAV infection, mock infection and PBMC-only). *Post-hoc* tests were done using the *t*-test corrected method for multiple testing, with the method of Holm. Comparisons between 24 and 48 h of co-culture were done using the non-paired Mann Whitney *U*-test. Two-tailed *p*-values less than 0.05 were considered statistically significant.

## Results

### Co-culture model of H3N2-infected hNEC and PBMC

hNECs were derived from hNESPCs isolated from biopsies of a non-smoker asthma patient (Table 1A). The co-culture model of H3N2 IAV-infected hNEC and PBMC from healthy donors (Table 1B) was established in transwell plates (Figure 1A). hNEC cells were seeded into the upper compartment, where they formed a complete multicellular epithelial layer after 24 days of culture. Infection of the epithelial layer was carried out 24 h prior to the addition of the PBMC; which were placed into the lower compartment of chamber. As the separation by the membrane of the transwell prevented direct contact with the epithelial layer, the model allowed the study of PBMC activation by soluble factors released from the virus-infected epithelial cells.

In order to exclude a direct infection of the PBMC by the virus, it was necessary to ensure the integrity of the epithelial layer. This was done by TEER measurements (Figure 1B). For this purpose, one insert for each culture condition (Mock and IAV-infected) was randomly selected at each time-point to act as control of epithelium integrity. The median baseline TEER before complete media apical treatment was 1890 Ω. There were no statistically significant differences between the baseline readings and 24 and 48 h post co-culture establishment in RPMI (*p* > 0.05, respectively, by non-paired Mann Whitney *U*-test) (Figure 1B). Similarly, pairwise comparisons were done between the baseline TEER readings and treatment of the nasal epithelium. There was a statistically significant difference in terms of TEER readings only between 48 h of IAV co-culture and baseline TEER, 24 h of IAV co-culture and 48 h of mock co-culture (respectively, *p* = 0.0286, *p* = 0.0286, and *p* = 0.0286, by non-paired Mann Whitney *U*-test) (Figure 1B). To further ensure that no virus had reached the lower chamber, we also performed qPCR for viral RNA (NS1 and M1) from basal medium from all samples with IAV infection at both 24 and 48 h after co-culture. Both NS1 and M1 RNA were only detectable in serial dilutions of the stock solution until a virus titer of 3 PFU/μL. Importantly, none of the medium samples from the lower compartment had any detectable NS1 or M1 viral RNA (data not shown). We also performed plaque assay of the apical chamber, which confirmed active infection of the hNECs (Figure 1C).

### Effect of IAV-infected nasal epithelium on cytokine and chemokine secretion

**Figure 2:**
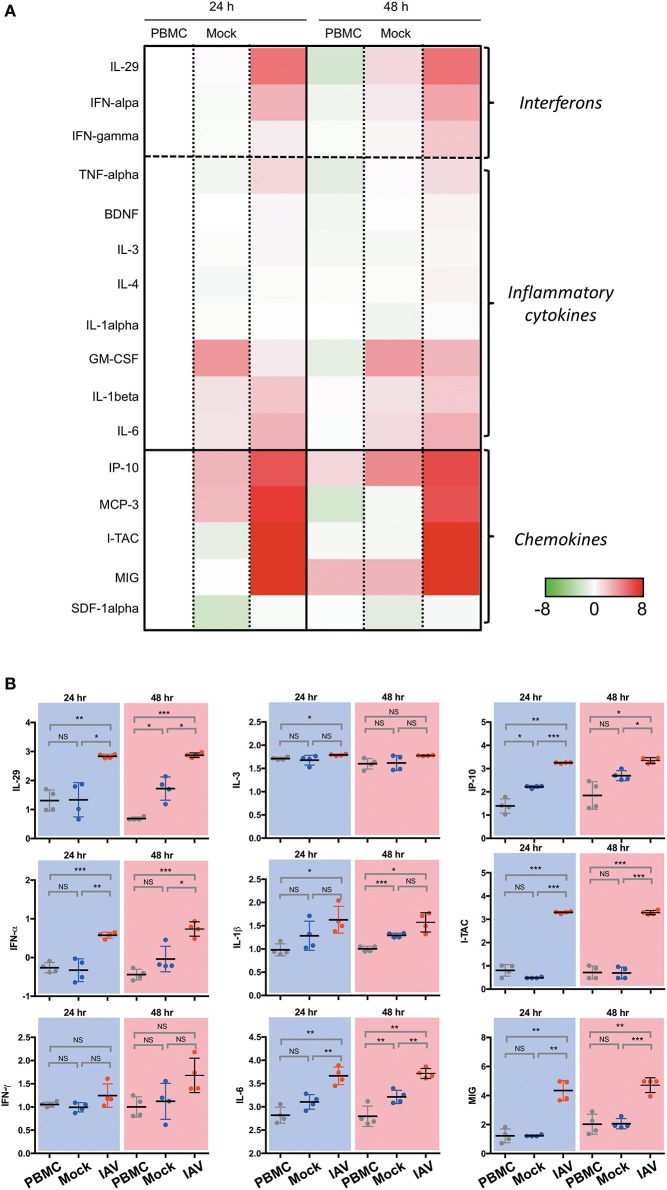
Luminex analysis of culture supernatants of PBMC, Mock, and IAV infection at 24 and 48 h. **(A)** Heatmap of interferon, selected cytokines and chemokines. The colors indicate the relative change (log2 fold change) of average analyte concentration in supernatant of PBMC, Mock, and IAV infection at 24 and 48 h compared to PBMC at 24 h. For each analyte, the relative concentration is shown as a scale from 8 and −8. **(B)** Statistically significantly upregulated cytokine and chemokine concentrations measured by Luminex multiplex assay on a custom 23-plex plate. Cytokines and chemokines are reported in log10 pg/ml. PBMC, peripheral blood mononuclear cell only sample; Mock, co-culture sample without H3N2-infection of nasal epithelium; IAV, co-culture of H3N2-infected nasal epithelium. All data represent the mean ± SD. ^***^P < 0.001; ^**^P < 0.01; ^*^P < 0.05; NS, not significant.

In a prior study, we have shown that epithelial cultures derived from hNESPCs responded to virus infection by the release of a number of cytokines and chemokines (7). To determine the composition of inflammatory cytokines and chemokines in the co-cultures, we analyzed the supernatants from the basal media taken 24 h and 48 h after co-culture establishment by Luminex. The experiments were carried out independently with PBMC of four donors. The heat maps in Figure 2A depict the relative change in their concentrations in one of these donors with reference to the respective PBMC 24 h value. The complete dataset is depicted in Supplementary Figure 3, the absolute values of selected cytokines and chemokines are shown in Figure 2B.

In line with expectations, the IAV samples show a clear increase in Type I- (IFN-α), Type II- (IFN-γ), and Type III-interferons (IL-29). For the members of all three interferon types, the upregulation in the infected sample was evident both at 24 and 48 h. The cytokine release was strongly associated with the infected state, as interferon-levels in the PBMC and Mock control cultures remained low. A similarly clear signature was also evident for TNF-α, and, to a weaker extent, for BDNF, IL-3 and IL-4. While a significant upregulation was detected for IL-6 and IL-1β, The effect was apparently not specific for the virus infection, as it was also evident in the mock-infected co-culture, albeit lower than the influenza-infected group.

The strongest increase was detected for chemokines. In particular, the levels of I-TAC (CXCL11), IP-10 (CXCL10), MIG (CXCL9), and MCP-3 (CCL7) increased nearly 6-fold upon infection. Very little or no infection-related effects were detected for SDF-1α (CXCL12) and IL-8 (CXCL8). As IP-10 is induced by IFN-γ, the increase of this chemokine is consistent with the observed upregulation of IFN-γ. Notably, both IP-10 and MCP-3 were reported to orchestrate lung inflammation (10) and influence asthma-related rhinovirus infections (11). Likewise, CXCL9 and CXCL11 have been associated with infections such as rhinovirus (12) and influenza virus (13).

### Effect of IAV-infected nasal epithelium on PBMC and NK activation

CD38 and CD69 are PBMC activation markers that can be directly assessed by flow cytometry. Using these markers, we studied the effect of influenza-infected epithelial cells on the various immune cell populations co-cultured with virus-infected epithelia cells (IAV) (Supplementary Figures 4A, B). The analysis covered NK cells and monocytes, innate T cells (MAIT, Vδ1, and Vδ2 γδ T cells) and conventional T cells (CD4+ and CD8+). Flow cytometry measurements were made from samples taken 24 and 48 h after addition of the PBMC. The gating strategy is depicted in Supplementary Figure 1.

**Figure 3:**
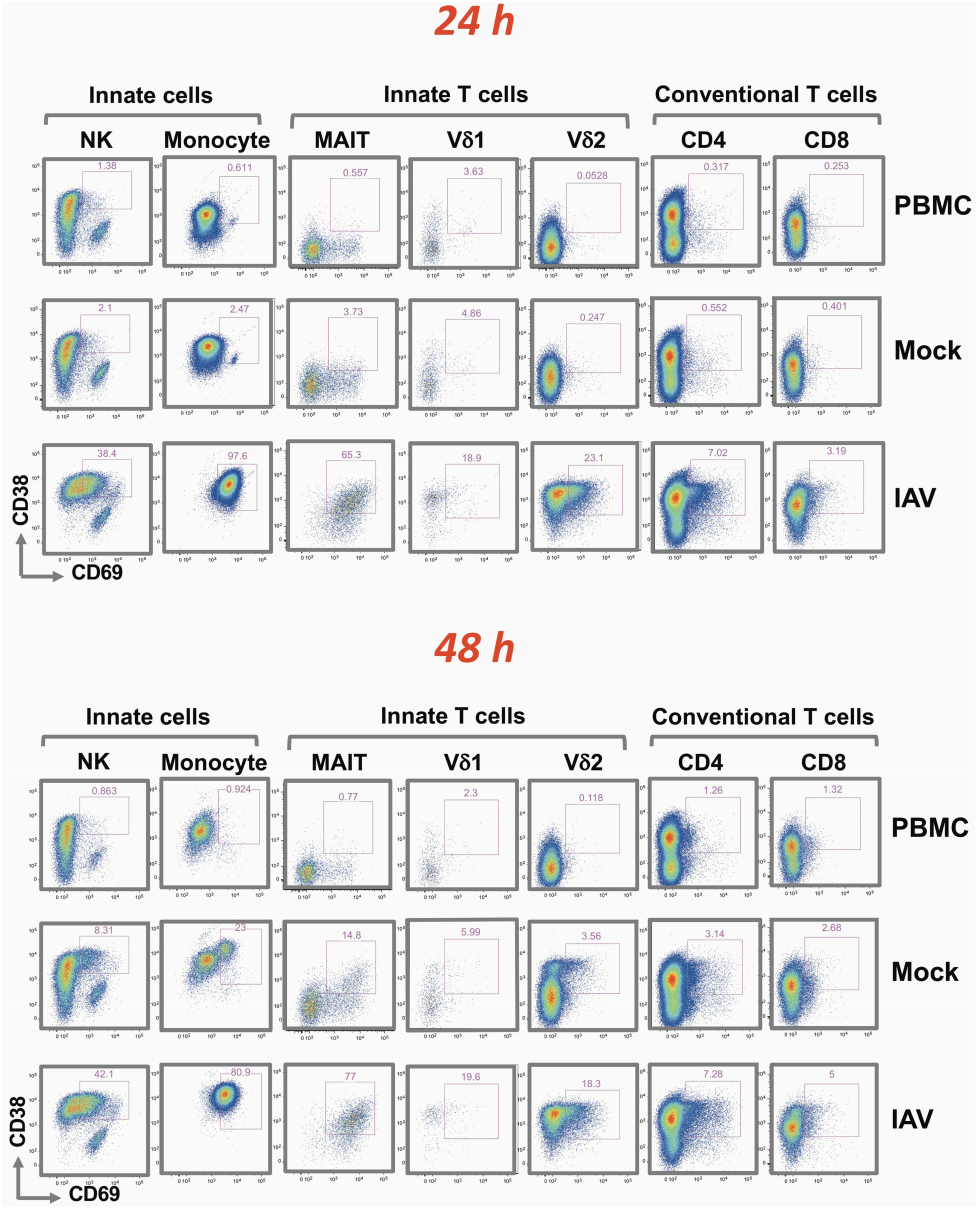
Rapid up-regulation of activation markers by mononuclear cells following H3N2 infection of nasal epithelium. Representation of flow cytometric analysis of CD38 and CD69 up-regulation in NK, monocyte, MAIT, Vδ1, Vδ2 T cells, CD4+, and CD8+ T cells for PBMC only, uninfected epithelium with PBMC, and influenza-infected epithelium with PBMC cultured for 24 and 48 h.

Although the activation of each PBMC subset was indicated by an increase in the fluorescence signal of CD38 and/or CD69, the extent of the shifts varied depending on the cell type (indicated in Figure 3 by cell type-specific activation gates). At 24 h, there were no apparent differences in terms of activation between the PBMC alone and Mock infected cultures. A very similar activation pattern was also observed after 48 h; with slight activation observed in the mock-infected culture. While IAV infection did not alter the frequency of the different immune cell types (Supplementary Table 1), it resulted in a clear increase of activation markers in all analyzed cell subsets. After 24 h, the entire monocyte population was activated. In CD4+ and CD8+ T cells, the fold increase in the mean fluorescence intensity (MFI) was rather modest. In line with the clonal nature of the adaptive arm, it affected only a small subset of the respective T cell population. It was more pronounced for the CD4+ cells, and increased only marginally after 48 h. The activation was substantially greater on NK cells and on innate T cells. In contrast to conventional T cells, for NK cells, MAIT and γδ T cells, the IAV-induced activation was evident in the majority of these subpopulations; similarly to the monocyte subset, activation was achieved already after 24 h. Particularly notable is the strong and quantitative activation of the MAIT cells and CD16+ CD56 dim NK cells in response to IAV (Supplementary Figure 2). While their T cell receptor is specific for bacterially-produced vitamin B metabolites (14), a recent study demonstrated that the subset is activated after influenza infection and is very effective in the viral clearance (15).

## Discussion

We performed this study to better understand the dynamics of early intranasal seasonal H3N2 IAV infection and innate immune responses. This could prove to be a crucial area of study in the development of diagnostic tools and preventive therapies for severe influenza infection. Up to one third of IAV-infected patients have been shown to be asymptomatic nasal carriers of IAV (16). Mechanisms that underlie an early protective innate immune response are poorly understood. NK cells have been already shown to be early and important factors during influenza infection (17, 18) and other studies have implicated a role of unconventional innate immune T cells in some viral infections. Protective early innate immune responses may be a promising target in the future for influenza prevention and pharmacotherapy.

Based on this study the co-culture system represents an excellent model to investigate specifically the early crosstalk between the infected epithelial layer and the cellular components of the innate arm of the immune response. After 24 h, complete activation is not only observed in monocytes; but also in NK cells, particularly the CD16+ CD56 dim population and innate T cell populations such as γδ and MAIT cells, all of which are cell types implicated in various viral infections. Similarly to innate cells, MAIT cells and γδ T cells represent the first line of defense against microbial infections (19, 20). For instance, MAIT cells have been shown to respond during influenza, dengue, and hepatitis infection (15). γδ T cells, which can be broadly divided into Vδ1 and Vδ2 T cells, play important roles in immune responses against microbial and non-microbial stresses. Whereas Vδ2 T cells respond to bacterial infections through reactivity to prenyl antigens (21), Vδ1 T cells have been implicated in viral clearance of infections caused by cytomegalovirus (22) and Epstein Barr virus (23) as well as a number of transformed cell lines (24) through secretion of IFN-γ. Influenza has also been documented to be able to directly infect and induce activation of NK cells, while NK cell depletion has been implicated in delayed influenza virus clearance (17).

It is important to stress that the upregulation of CD38+ and CD69+ on the PBMC subsets was not caused by direct H3N2 infection of these cells. The epithelial layer remained intact throughout the experiment, and qPCR did not detect viral RNA in the lower compartment of the co-culture harboring the PBMC. Also, the epithelium was separated from the PBMC by a Transwell insert, acting as a physical barrier preventing activation through direct contact between the cells and preventing activation due to HLA-related differences. Based on the cytokines and chemokines secreted by the co-culture, significantly increased levels of type I, II and III interferons were observed already after 24 h of co-culture. These could play a key role in the activation of the PBMC subpopulations. Exosomes secreted from the epithelium have also been implicated in cell activation signaling, and play important roles in cell-to-cell crosstalk (25). However, we were unable to single out the exosomic signatures using the current setting, and thus further scaling up of the model can be explored in the future to investigate the role of exosomes in cell activation. While the impact of type III interferon (IL-29) is not known; a previous study showed that type III interferons clearly contribute to the activation of MAIT cells (13). While the release of interferons could be a result of the T cell interaction with the activated monocyte subset, we have shown in a prior study that all three types of interferon are produced by the infected epithelial cells (7). This raises the intriguing question whether the interferons and other soluble factors released by the epithelial cell layer alone are sufficient to drive the activation of innate T and NK cells; as well as the role of monocytes in this activation process. While these questions can be addressed in future studies using the co-culture system, it also provides the tools to identify the specifc factors responsible for their activation.

In contrast to NK and innate T cells, only a small fraction of conventional T cells became activated. The numbers were higher on CD4+ T cells in comparison to CD8+ T cells. Activation of CD4+ T cells after 24 h of co-culture, as evidenced by upregulation of CD69 is in line with previous studies describing early recruitment of CD4+ T cells in response to IAV and IAV vaccination (26, 27). CD4+ T cells have also been reported to be the major T cell component in the antiviral response, in order to orchestrate recruitment of dendritic cells and to mount the adaptive immune responses of B cells and CD8+ T cells (28). Activation of unconventional innate immune T cells and monocytes, in turn, occurs at an early stage and there were few significant differences between 24 and 48 h of co-culture in the *post-hoc* analyses. Higher levels of CD38 associated with Vδ2 T cells and monocytes, whereas higher levels of CD69 seemed to associate with MAIT and NK cells. Notably, the majority of the respective population subsets became activated upon infection without direct contact with the virus.

Significantly increased local production of IL-6 intranasally during IAV infection of the nose has been documented previously in a clinical study (29). Interestingly, increased plasma levels of IL-6 have been implicated in severe cases of pandemic H1N1 infection resulting in admission into the critical care unit and predicted a fatal outcome (30). Another study involving organ transplant patients vaccinated against seasonal influenza A virus identified that patients with both seroconversion and seroprotection had significantly elevated levels of IFN-α, IFN-γ, MCP-3, IL-1β, IP-10, IL-2, IL-4, IL-5, MCP-1, and TNF-α in PBMC supernatants (31), which match most of our cytokines and chemokines with highest concentrations. Interestingly, we observed high concentrations of the same cytokines as did another group in the 72 h post-inoculation nasal washes in a study on patients using intranasally administered LAIV (32). They observed IP-10 and MCP-1 in all subjects and G-CSF in 84% subjects, whilst IFN-α2 was only detectable in 34% of subjects (32).

This study was undertaken to provide *in vitro* information on the events taking place intranasally during H3N2 IAV infection. Based on the literature, we can already observe a correlation between our results and those of clinical studies involving both nasal wash samples and serum samples, even though we used a small number of donors. This would imply that our results are partly congruent with *in vivo* conditions. The limitations of our study include the small number of nasal epithelium and PBMC donors. We did not study all immune cells when studying PBMCs or exosomic signals, but based on the focus of our study on the early crosstalk, it is sufficient.

Our model's strengths are that it provides a study platform that seems to be partly congruent with clinical settings, enabling development and testing of better targeted and effective novel drugs. Further studies are needed with direct clinical replication of our results involving a larger number of patients.

## Conclusion

Currently, *in vitro* influenza infection models mimicking *in vivo* situations are lacking. We were able to establish a co-culture with epithelium integrity preserved after H3N2 infection of nasal epithelium. Our results are apparently congruent with studies performed *in vivo* intranasally. In the future, it would be important to replicate our results *in vivo* in patients presenting with seasonal H3N2 influenza virus and compare those results with our current *in vitro* results. We would also seek to combine the current model with a more complex *in vitro* model that includes cell-cell interactions to fully elucidate antiviral immune responses *in vitro*. Our model could be used to evaluate efficacy of new antiviral and immunomodulatory drugs targeting innate immune pathways, in order to ameliorate severe influenza infection.

## Author contributions

All authors participated in the study plan. KP, NY, KT, YY, JL, VC, OR, and DW made the applications. AL, KP, NY, KT, YY, JL, VC, OR, and DW recruited the subjects. AL, KP, KT, YY, and JL conducted all experiments. AL, KP, NY, BL, KT, ST-S, RR, and OR conducted data management and performed statistical testing. All authors participated in manuscript writing and critical review.

### Conflict of interest statement

The authors declare that the research was conducted in the absence of any commercial or financial relationships that could be construed as a potential conflict of interest.

## Supplementary material

Supplementary Figure 1 Flow cytometry gating strategy.

Supplementary Figure 2 Flow cytometric analysis of NK cell sub-populations after co-culture with influenza-infected epithelium. Gating strategy for NK cells in PBMC **(A)**. CD38 up-regulation in CD16+ CD56dim (pink), CD16- CD56dim (light blue), and CD16- CD56hi (dark blue) NK cells after PBMC co-culture with influenza-infected epithelium for 24 h **(B)**. Gating strategy for purified NK cells **(C)**. CD38 up-regulation in CD16+ CD56dim (pink), CD16- CD56dim (light blue), and CD16- CD56hi (dark blue) after isolated NK cells co-cultured with influenza-infected epithelium for 24 h. Values in the histograms are the geometric mean of CD38 expression level.

Supplementary Figure 3 Heatmap of the 23 analytes measured by Luminex at 24 h and 48 h for PBMC, uninfected epithelium with PBMC (Mock), and influenza-infected epithelium with PBMC (IAV). The colors indicate the relative change (log2 fold change) of analyte concentration from each donor in the supernatant of PBMC, Mock, and IAV infection at 24 h and 48 h compared to PBMC at 24 h. For each analyte, the relative concentration is shown as a scale from 15 and −10.

Supplementary Figure 4 Summary of CD38 and CD69 upregulation of various immune cells measured by flow cytometry for PBMC only, uninfected epithelium with PBMC, and influenza-infected epithelium with PBMC cultured for 24 h **(A)** and 48 h **(B)**. All data represent the mean ±SD. ^***^, *P* < 0.001; ^**^, *P* < 0.01; ^*^, *P* < 0.05; ns, not significant.

Supplementary Table 1 Table of mononuclear cell population frequencies following H3N2 infection, mock infection, and in PBMC-only.

## Abbreviations

hNEC: human nasal epithelial cell
hNESPC: human nasal epithelium stem/progenitor cell
IAV: influenza A virus
IL-13: Interleukin 13
γδ T cells: gamma delta T cell
LAIV: Live attenuated influenza virus
MAIT: mucosal associated invariant T cell
M1: matrix gene
NK cell: Natural Killer cell
NS1: non-structural gene
PBMC: peripheral blood mononuclear cells
PFU: plaque forming unit
SPT: skin prick test
TCR: T cell receptor
TEER: transepithelial electrical resistance.
