# Supplementary figures and images for: A Co-culture Model of PBMC and Stem Cell Derived Human Nasal Epithelium Reveals Rapid Activation of NK and Innate T Cells Upon Influenza A Virus Infection of the Nasal Epithelium
Source: Front Immunol. 2018 Nov 8;9:2514. doi: 10.3389/fimmu.2018.02514 (PMC6237251; doi:10.3389/fimmu.2018.02514)

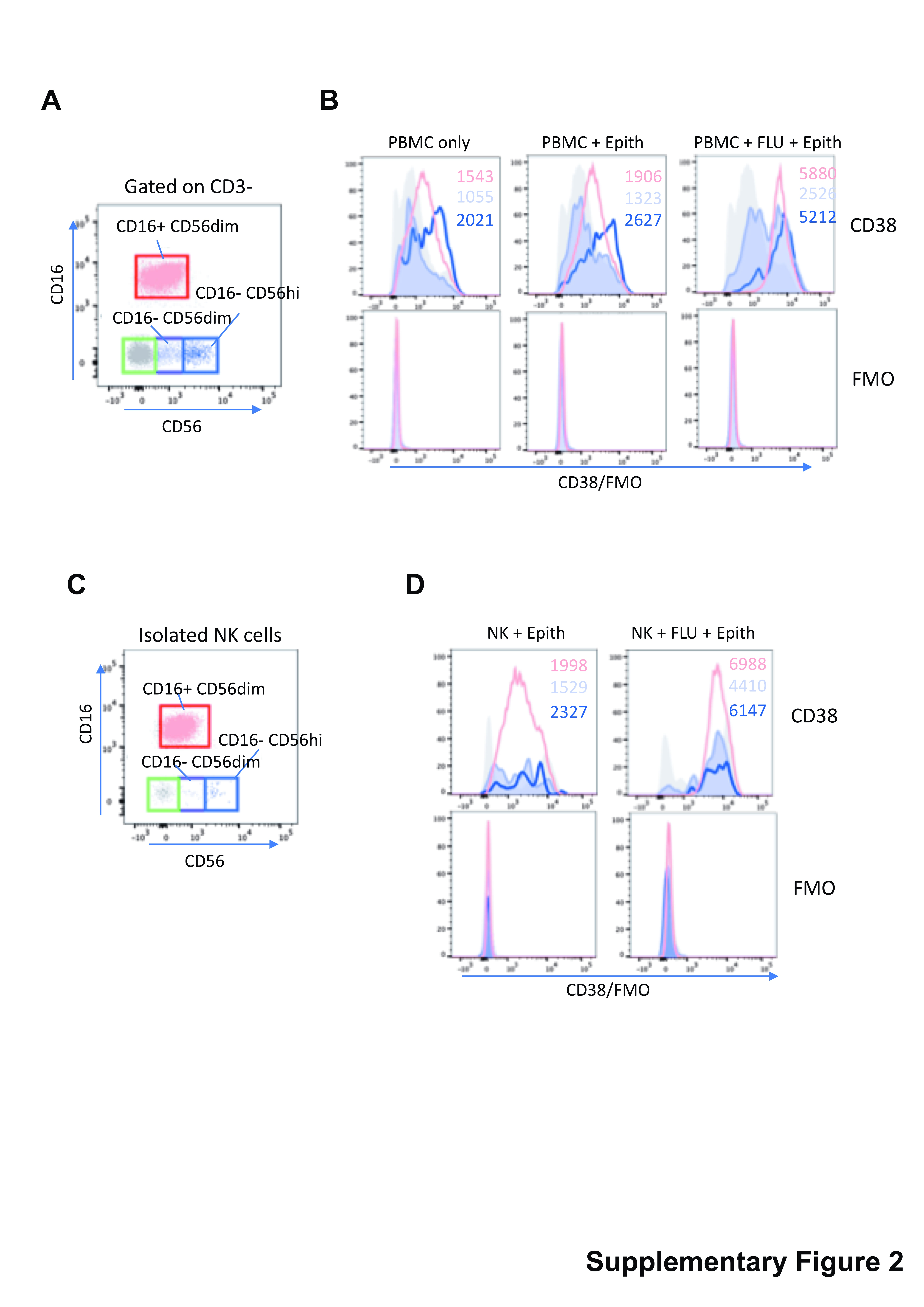

Supplement: Supplementary Figure 2 — Flow cytometric analysis of NK cell sub-populations after co-culture with influenza-infected epithelium. Gating strategy for NK cells in PBMC (A). CD38 up-regulation in CD16+ CD56dim (pink), CD16- CD56dim (light blue), and CD16- CD56hi (dark blue) NK cells after PBMC co-culture with influenza-infected epithelium for 24 h (B). Gating strategy for purified NK cells (C). CD38 up-regulation in CD16+ CD56dim (pink), CD16- CD56dim (light blue), and CD16- CD56hi (dark blue) after isolated NK cells co-cultured with influenza-infected epithelium for 24 h. Values in the histograms are the geometric mean of CD38 expression level. [file Image_2.jpg]
