# Supplementary material for: A Co-culture Model of PBMC and Stem Cell Derived Human Nasal Epithelium Reveals Rapid Activation of NK and Innate T Cells Upon Influenza A Virus Infection of the Nasal Epithelium
Source: Front Immunol. 2018 Nov 8;9:2514. doi: 10.3389/fimmu.2018.02514 (PMC6237251; doi:10.3389/fimmu.2018.02514)
Supplement: Supplementary Table 1 — Table of mononuclear cell population frequencies following H3N2 infection, mock infection, and in PBMC-only. [file Table_1.pdf]

Donor	Treatment	Time	CD8 Freq of CD3	MAIT Freq of CD3	CD4 Freq of CD3	Vd1 Freq of CD3	Vd2 Freq of CD3	Nk Freq of Lymh	mono Freq of LIVE
Donor 1	PBMC	24 h	19,7	3,15	62	0,547	22,4	9,43	19,4
Donor 1	Mock	24 h	19,5	3,11	61,8	0,534	21,8	10,3	21,4
Donor 1	IAV	24 h	20,1	2,8	62,8	0,498	21,5	9,91	24,4
Donor 2	PBMC	24 h	29,3	1,48	64,3	0,704	4,5	4,45	9,38
Donor 2	Mock	24 h	29,8	1,58	63,7	0,686	4,59	4,41	8,66
Donor 2	IAV	24 h	29,3	1,45	64,6	0,734	4,37	5,13	10,3
Donor 3	PBMC	24 h	22,1	7,57	60,1	1,3	18,7	8,99	12,8
Donor 3	Mock	24 h	23,4	10,6	56,9	1,07	8,26	8,93	15,9
Donor 3	IAV	24 h	22,8	8,02	58,8	1,11	10,7	8,99	16,6
Donor 4	PBMC	24 h	28,6	8,78	52,9	0,855	8,66	10,2	13,6
Donor 4	Mock	24 h	29,6	9,65	51,7	0,835	7,76	13	15,5
Donor 4	IAV	24 h	29,5	8,94	52,3	0,829	6,42	14,4	17,7
Donor 1	PBMC	48 h	17,8	2,84	64,4	0,581	22,3	11,6	2,74
Donor 1	Mock	48 h	18	2,8	64,2	0,519	23,2	11,2	7,65
Donor 1	IAV	48 h	17,9	2,51	65,7	0,371	22,9	10,7	17,7
Donor 2	PBMC	48 h	28,4	1,42	65,4	0,628	4,46	4,66	1,75
Donor 2	Mock	48 h	29,3	1,55	64,1	0,596	4,69	4,61	3,76
Donor 2	IAV	48 h	26,3	0,857	68,1	0,851	2,93	3,04	1,56
Donor 3	PBMC	48 h	22,1	9,68	59,3	0,996	7,29	8,75	4,02
Donor 3	Mock	48 h	23,6	10,5	56,6	0,907	7,86	8,35	2,08
Donor 3	IAV	48 h	22,6	8,16	58,2	0,92	7,61	9,03	10,3
Donor 4	PBMC	48 h	28,2	8,25	55	0,802	5,51	13,6	2,69
Donor 4	Mock	48 h	30,1	8,85	52,1	0,722	5,71	14,6	3,44
Donor 4	IAV	48 h	30,6	7,8	52,2	0,822	5,5	15,1	12,7

Supplementary Table 1
